# Uses of Cyanoacetylhydrazine in Heterocyclic Synthesis: Novel Synthesis of Pyrazole Derivatives with Anti-tumor Activities

**DOI:** 10.3390/molecules17078449

**Published:** 2012-07-12

**Authors:** Rafat M. Mohareb, Nahed N. E. El-Sayed, Mahmoud A. Abdelaziz

**Affiliations:** 1 Departrment of Chemistry, Faculty of Science, Cairo University, Giza 12613, Egypt; 2 Department of Organic Chemistry, Faculty of Pharmacy, October University for Modern Sciences & Arts (MSA), El-Wahaat Road, October City, Giza 12411, Egypt; 3 National Organization for Drug Control and Research, P.O. Box 29, Cairo 11421, Egypt; 4 College of Science, Department of Chemistry, King Saud University, Riyadh 11932, Saudi Arabia; 5 Preparatory Year Department, AL-Ghad International Colleges for Health Sciences, Tabuk Male, Tabuk 41321, Saudi Arabia; 6 Basic Science Department, Modern Academy for Engineering and Technology in Maadi, Maadi 11431, Cairo, Egypt

**Keywords:** **:** pyrazole, thiophene, thiazole, antitumor

## Abstract

The reaction of cyanoacetylhydrazine with chloroacetyl chloride gave *N'*-(2-chloroacetyl)-2-cyanoacetohydrazide. The latter underwent cyclization to afford 1-(5 amino-3-hydroxy-1*H*-pyrazol-1-yl)-2-chloroethanone, which underwent nucleophilic substitution to give 3-(5-amino-3-hydroxy-1H-pyrazol-1-yl)-3-oxopropanenitrile. The latter two compounds were used as key synthons to synthesize new thiophene, pyran, thiazole and some fused heterocyclic derivatives. The antitumor activity of the newly synthesized compounds was evaluated against three human tumor cells lines, namely breast adenocarcinoma (MCF-7), non-small cell lung cancer (NCI-H460) and CNS cancer (SF-268) and some of these compounds were found to exhibit much higher inhibitory effects towards the three tumor cell lines than the Gram positive control doxorubicin.

## 1. Introduction

Cancer is a major public health problem in the world. Chemotherapy is still one of the primary modalities for the treatment of cancer. However, the use of this method is limited mainly due to the small number of the available chemotherapeutic agents to choose among them and also because the use of these agents is often accompanied by undesirable side effects. This clearly underlies the urgent need for developing novel chemotherapeutic agents with more potent antitumor activities and reduced side effects.

Many pyrazole derivatives have attracted considerable attention in the recent years for their diverse biological activities [1,2,3,4,5,6]. They are also acknowledged for their anticancer activities [7,8,9]. Moreover, the chemistry of fused pyrazolo- and thieno-pyrazole derivatives has drawn great attention due to their pharmacological importance [10,11,12]. Such excellent pharmacology encouraged us to synthesize novel pyrazole derivatives with evaluation of their antitumor activities.

## 2. Results and Discussion

### 2.1. Chemistry

The starting material, *N'*-(2-chloroacetyl)-2-cyanoacetohydrazide (**3**) was prepared by reacting cyanoacetylhydrazine (**1**) with chloroacetyl chloride (**2**) in 1,4-dioxane. Structural elucidation of compound **3** was based on its ^1^H-NMR and ^13^C-NMR data. Thus, the ^1^H-NMR spectrum showed the presence of two singlets at δ 3.51, 4.05 ppm indicating the presence of the two CH_2_ groups and two broad singlets at δ 8.22–8.26 ppm corresponding to the two NH groups. Moreover, the ^13^C-NMR spectrum revealed the presence of the following signals at δ: 25.8, 40.8 (2 CH_2_), 116.8 (CN), 162.3, 168.9 (2 C=O).

**Scheme 1:**
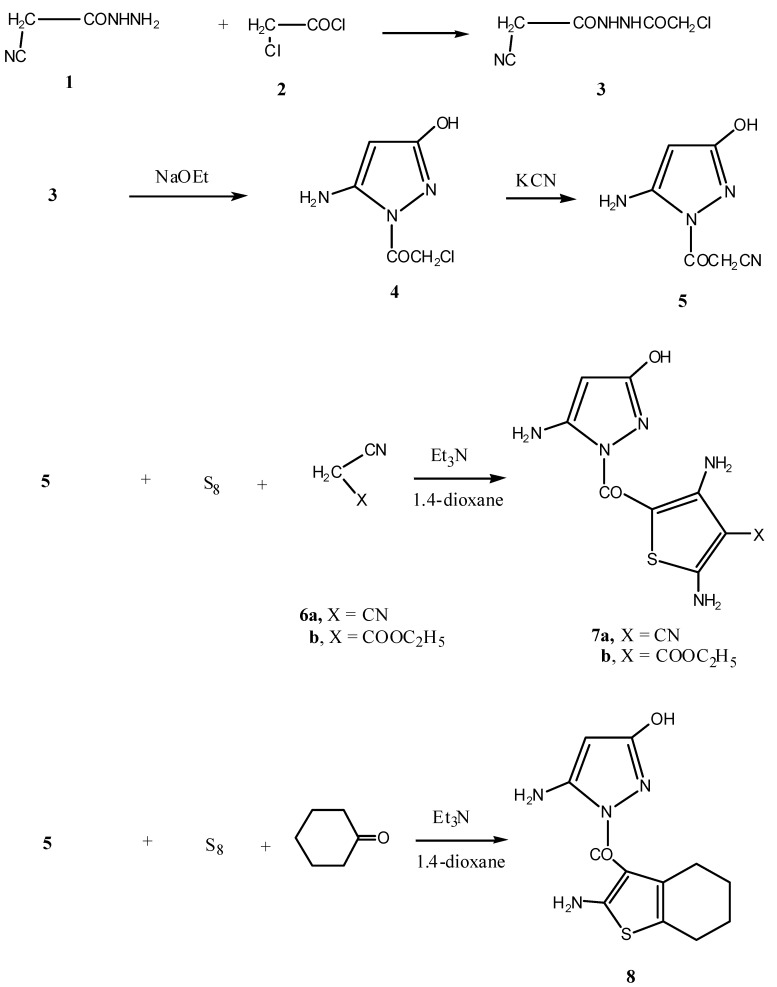
Synthesis of compounds **3**, **5**, **7a**,**b** and **8**.

Heterocyclization of *N*-α-halocarbonyl derivatives using sodium ethoxide has been previously reported [13,14,15,16,17]. Thus, compound **3** readily underwent cyclization when heated in sodium ethoxide solution to give 1-(5-amino-3-hydroxy-1*H*-pyrazol-1-yl)-2-chloroethanone (**4**). The IR spectrum of this compound indicated the presence of OH and NH_2_ groups at 3583–3305 cm^−1^ and C=O at 1693 cm^−1^. Furthermore the ^1^H-NMR spectrum revealed the presence of a singlet δ at 4.51 ppm, a singlet at δ 4.88 ppm, a singlet at δ 6.89, and a singlet at δ 10.36 ppm (D_2_O exchangeable) corresponding to the CH_2_, NH_2_, the pyrazole H-4 and the OH group protons, respectively. Compound **4** was converted to the corresponding *N*-carbonylacetonitrilopyrazole derivative **5** by nucleophilic substitution of the chlorine atom using potassium cyanide. Subjecting compound **5** to the Gewald thiophene synthesis [18,19,20,21,22] via its reaction with either malononitrile (**6a**) or ethyl cyanoacetate (**6b**) and elemental sulfur in presence of triethylamine as basic catalyst afforded the pyrazol-1-yl *N*-thiophen-5-yl derivatives **7a** and **7b**, respectively. On the other hand, the reaction of compound **5** with cyclohexanone and elemental sulfur in presence of triethylamine as basic catalyst afforded the (5-amino-3-hydroxy-1*H*-pyrazol-1-yl)(2-amino-4,5,6,7‑tetrahydrobenzo-[*b*]thiophen-3-y0,l)methanone derivative **8** (Scheme 1). The analytical and spectroscopic data of compounds **7a**,**b** and **8** are consistent with the proposed structures (see Experimental section). Thus, the ^1^H-NMR spectrum of compound **8** showed the presence of two multiplets at δ 2.23–2.27 ppm indicating the presence of the four CH_2_, two singlets at δ 4.49, 4.80 ppm indicating the presence of two NH_2_ groups, a singlet at δ 6.89 ppm indicating the pyrazole H-4 and a singlet at δ 10.23 ppm corresponding to the OH group. Moreover, the ^13^C-NMR showed the following signals at δ: 20.0, 23.1, 14.0, and 24.6 (cyclohexane C), 109.3, 113.4, 138.6, 143.8, 144.8, 150.2, and 152.9 (pyrazole, thiophene C), 160.6 (C=O).

**Scheme 2:**
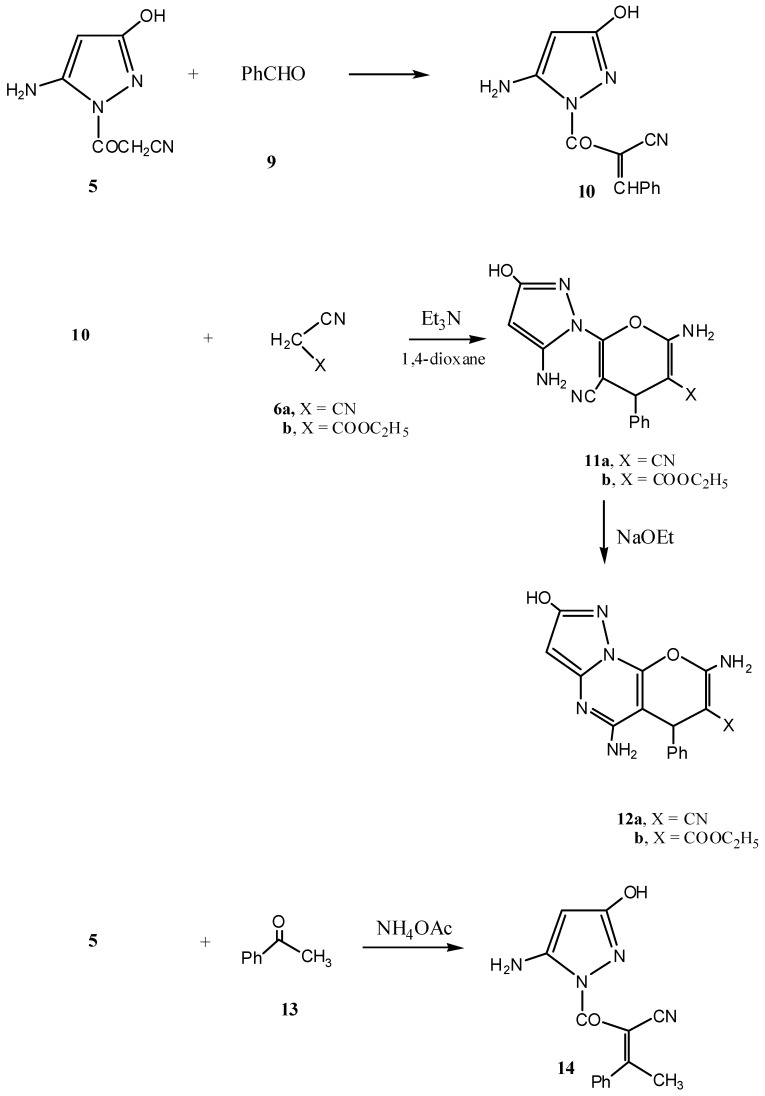
Synthesis of compounds **10**, **11a**,**b**, **12a**,**b** and **14**.

The reaction of compound **5** with benzaldehyde (**9**) gave the phenylmethylidene derivative **10**. The latter showed interesting reactivity towards cyanomethylene reagents, namely malononitrile (**6a**) and ethyl cyanoacetate (**6b**) and afforded the pyrazole-1-yl-pyran derivatives **11a** and **11b**, respectively. The latter products underwent ready cyclization in sodium ethoxide solution to give thedihydropyrazolo[1,5-*a*]pyrano[2,3-*d*]pyrimidines **12a** and **12b**, respectively. On the other hand, heating compound **5** with acetophenone (**13**) in an oil bath at 120 °C in the presence of ammonium acetate afforded the Knoevenagel condensation product **14** (Scheme 2) [23,24,25,26,27].

**Scheme 3:**
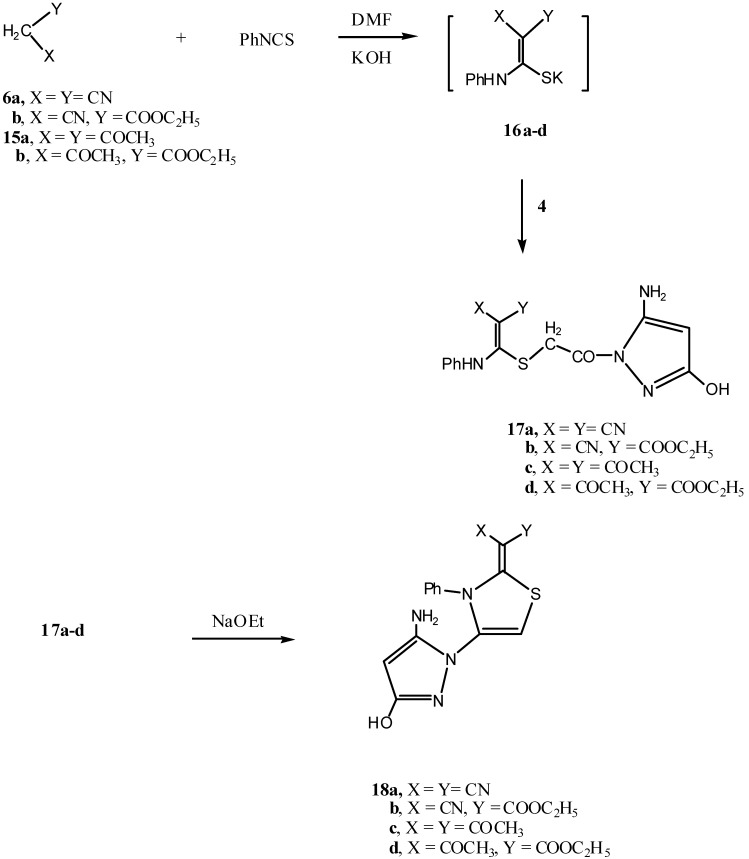
Synthesis of compounds **17a**–**d** and **18a**–**d**.

Finally, the reactivity of compound **4** as an α-halocarbonyl compound to produce thiazole derivatives was investigated. Thus, the reaction of the active methylene reagents **6a**,**b** and **15a**,**b** with phenylisothiocyanate in DMF/KOH solution afforded the nonisolable intermediate potassium sulfide salts **16a**–**d** which in turn were allowed to react *in situ* with compound **4** to form the thioether derivatives **17a**–**d**, respectively. The analytical and spectroscopic data of the latter products were in agreement with the assigned structures. Compounds **17a**–**d** underwent ready cyclization when heated in sodium ethoxide solution to give the pyrazol-1-yl-thiazole derivatives **18a**–**d**, respectively (Scheme 3). The structures of compounds **18a**–**d** were confirmed on the basis of their respective ^1^H-NMR and ^13^C-NMR spectra (see Experimental section).

### 2.2. Antitumor Evaluations

#### 2.2.1. Structure Activity Relationship of the Newly Synthesized Products

The effect of the newly synthesized products was evaluated on the *in-vitro* growth of three human tumor cell lines representing different tumor types, namely, breast adenocarcinoma (MCF-7), non-small cell lung cancer (NCI-H460) and CNS cancer (SF-268), after a continuous exposure of 48 h. The results are summarized in Table 1.

**Table 1:** Effect of the newly synthesized compounds on the growth of three human tumor cell lines.

Compound	GI_50_ (mol L^−1^)		
	MCF-7	NCI-H460	SF-268
**3**	20.1 ± 0.6	16.3 ± 1.4	22.3 ± 1.5
**4**	22.6 ± 0.4	21.3 ± 0.8	22 ± 0.8
**5**	40.6 ± 16.9	38.9 ± 10.8	20.8 ± 8.6
**7a**	40.6 ± 12.2	32.6 ± 8.6	60.4 ± 14.8
**7b**	35.4 ± 8.2	26.1 ± 2.8	28.9 ± 4.8
**8**	11.8 ± 0.6	14.5 ± 0.8	16.7 ± 1.6
**10**	33.7 ± 17.5	20.2 ± 8.8	12.0 ± 2.4
**11a**	2.1 ± 0.7	1.2 ± 0.8	1.4 ± 0.8
**11b**	20.0 ± 1.2	20.6 ± 3.4	18.4 ± 2.6
**12a**	0.01 ± 0.001	0.01 ± 0.008	0.02 ± 0.001
**12b**	16.0 ± 3.6	20.0 ± 2.4	18.5 ± 6.0
**14**	50.6 ± 12.9	36.4 ± 8.8	44.8 ± 6.6
**17a**	0.1± 0.02	0.4 ± 0.01	0.4 ± 0.08
**17b**	12.4 ± 8.2	10.1 ± 2.8	8.2 ± 1.8
**17c**	6.2 ± 1.6	4.2 ± 1.8	2.7 ± 0.6
**17d**	0.2 ± 0.01	0.1 ± 0.06	0.3 ± 0.05
**18a**	0.02 ± 0.008	0.03 ± 0.008	0.01 ± 0.004
**18b**	20.0 ± 3.6	22.0 ± 2.4	31.5 ± 8.0
**18c**	0.03 ± 0.006	0.01 ± 0.006	0.03 ± 0.005
**18d**	1.9 ± 0.9	0.6 ± 1.8	0.8 ± 0.08
Doxorubicin	0.04 ± 0.008	0.09 ± 0.008	0.09 ± 0.007

Results are given in concentrations that were able to cause 50% of cell growth inhibition (GI_50_) after a continuous exposure of 48 h and show means ± SEM of three-independent experiments performed in duplicate

All the compounds were able to inhibit the growth of the human tumor cell lines in a dose-dependent manner. 5,8-Diamino-7-cyano-2-hydroxy-6-phenyl-6,7-dihydro−[1,5−α] pyrano[2,3-*d*] pyrimidine(**12a**), 2-(4-(5-amino-3-hydroxy-1*H*-pyrazol-1-yl)-3-phenylthiazol-2(*3H*)-ylidene)-malononitrile (**18a**) and 2-(4-(5-amino-3-hydroxy-1*H*-pyrazol-1-yl)-3-phenylthiazol-2(*3H*)-ylidene) pentane-2,4-dione (**18c**) showed the best results, exhibiting the highest inhibitory effects of the tested compounds towards the three tumor cell lines, which are higher than that of the reference compound doxorubicin. On the other hand, compounds **17a**, **17d**, and **18d** showed high growth inhibitory effect but such activity are lower than the reference, doxorubicin. Comparing the activities of **11a** and **11b** indicated that the presence of the CN group in **11a** resulted a stronger growth inhibitory effect than COOC_2_H_5_ group in compound **11b**. Similarly comparing the reactivity of compounds **12a** and **12b** indicated that the presence of the CN group in **12a** also is responsible of its higher reactivity over **12b**. On the other hand, compound **17a** with the CN group is the most active compound towards the three cancer cell lines among the pyrazole derivatives **17a**–**d**, of course the presence of the CN group in **17a** is responsible for such high activity. On the other hand, compound **17c** where X = Y = COCH_3_ is of lower activity than **17d** with X = COCH_3_ & Y = COOC_2_H_5_. Finally it worthy to refer to observation that indicated also that compound **18a** showed the highest reactivity towards the three cancer cell line among compound **8a**–**d**. On the other hand, compounds **5**, **7a**, **7b**, **10** and **14** exhibited the lowest reactivity towards the cancer cell lines.

## 3. Experimental

### 3.1. Antitumor Activity Tests

Reagents: Fetal bovine serum (FBS) and L-glutamine, were from Gibco Invitrogen Co. (Oxford, UK). RPMI-1640 medium was from Cambrex (Ashland, NJ, USA). Dimethyl sulfoxide (DMSO), doxorubicin, penicillin, streptomycin and sulforhodamine B (SRB) were obtained from Sigma Chemical Co. (St. Louis, MO, USA).

Cell cultures: Three human tumor cell lines, MCF-7 (breast adenocarcinoma), NCI-H460 (non-small cell lung cancer), and SF-268 (CNS cancer) were used. MCF-7 was obtained from the European Collection of Cell Cultures (ECACC, Salisbury, UK) and NCI-H460 and SF-268 were kindly provided by the National Cancer Institute (NCI, Cairo, Egypt). They grow as monolayer and routinely maintained in RPMI-1640 medium supplemented with 5% heat inactivated FBS, 2 mM glutamine and antibiotics (penicillin 100 U/mL, streptomycin 100 μg/mL), at 37 °C in a humidified atmosphere containing 5% CO_2_. Exponentially growing cells were obtained by plating 1.5 × 10^5^ cells/mL for MCF-7 and SF-268 and 0.75 × 10^4^ cells/mL for NCI-H460, followed by 24 h of incubation. The effect of the vehicle solvent (DMSO) on the growth of these cell lines was evaluated in all the experiments by exposing untreated control cells to the maximum concentration (0.5%) of DMSO used in each assay.

Tumor cell growth assay: The effects of compounds **3**–**18a**–**d** on the *in-vitro* growth of human tumor cell lines were evaluated according to the procedure adopted by the National Cancer Institute (NCI, Cairo, Egypt). In the ‘ *in-vitro* Anticancer Drug Discovery Screen’ that uses the protein-binding dye sulforhodamine B to assess cell growth [28]. Briefly, cells growing exponentially in 96-well plates were then exposed for 48 h to five serial concentrations of each compound, starting from a maximum concentration of 150 μM. Following this exposure period adherent cells were fixed, washed, and stained. The bound stain was solubilized and the absorbance was measured at 492 nm in a plate reader (Bio-Tek Instruments Inc., Powerwave XS, Wincoski, San Diego, CA, USA). For each test compound and cell line, a dose-response curve was obtained and the growth inhibition of 50% (GI_50_), corresponding to the concentration of the compounds that inhibited 50% of the net cell growth, was calculated as described elsewhere [29]. Doxorubicin was used as a positive control and tested in the same manner.

### 3.2. Chemistry

All melting points are uncorrected. IR spectra were recorded for KBr discs on a Pye Unicam SP-1000 spectrophotometer. ^1^H-NMR and ^13^C-NMR spectra were measured on a Varian EM-390–200 MHz in DMSO as solvent using TMS as internal standard, and chemical shifts are expressed as δ. Analytical data were obtained from the Microanalytical Data Unit at Cairo University, Giza, Egypt and the Microanalytical Data Unit at Erlangen University, Erlangen, Germany.

*N'-(2-Chloroacetyl)-2-cyanoacetohydrazide* (**3**). To a solution of cyanoacetylhydrazide (1.0 g, 0.01 mol) in 1,4-dioxane (20 mL), chloroacetylchloride (1.12 g, 0.01 mol) was added. The reaction mixture was stirred at room temperature overnight, then evaporated under vacuum. The residue was triturated with ethanol and the formed solid product was collected by filtration. Colorless crystals from ethanol, yield 1.40 g (80%), m.p. 135 °C. *Anal*. Calculated for C_5_H_6_ClN_3_O_2_ (175.57): C, 34.20; H, 3.44; N, 23.93. Found: C, 34.44; H, 3.29; N, 24.31. IR, υ: 3366–3238 (2NH), 2926 (CH_2_), 2256 (CN), 1688–1678 (2 CO). ^1^H-NMR, δ: 3.51, 4.05 (2s, 4H, 2CH_2_), 8.22–8.26 (2s, 2H, 2NH). ^13^C-NMR, δ: 25.8, 40.8 (2 CH_2_), 116.8 (CN), 162.3, 168.9 (2 CO).

*1-(5-Amino-3-hydroxy-1H-pyrazol-1-yl)-2-chloroethanone* (**4**). To a suspension of compound **3**(1.75 g, 0.01 mol) in sodium ethoxide solution [prepared by dissolving metallic sodium(0.64 g, 0.01 mol) in absolute ethanol (30 mL)] was heated in a boiling water bath for 3 h. The reaction mixture was left to cool then poured onto crushed ice containing few drops of hydrochloric acid. The formed solid product was collected by filtration. Crystallized from ethanol to give white crystals, yield 1.54 g (88%), m.p. 188–191 °C. *Anal*. Calculated for C_5_H_6_ClN_3_O_2_ (175.57): C, 34.20; H, 3.44; N, 23.93. Found: C, 33.93; H, 3.31; N, 24.17. IR, υ: 3583–3305 (OH, NH_2_), 1693 (CO), 1655 (C=N). ^1^H-NMR, δ: 4.51 (s, 2H, CH_2_), 4.88 (s, 2H, NH_2_), 6.89 (s, 1H, pyrazole H-4), 10.36 (s, 1H, OH). ^13^C-NMR, δ: 41.3 (CH_2_), 105.2, 150.1, 152.3 (pyrazole C), 170.3 (CO).

*3-(5-Amino-3-hydroxy-1H-pyrazol-1-yl)-3-oxopropanenitrile* (**5**). To a well stirred solution of compound **4** (1.75 g, 0.01 mol) in ethanol (30 mL) at 60 °C was added dropwise a solution of potassium cyanide (1.70 g, 0.02 mol in 5 mL water). Stirring was continued for 1 h and the resulting reaction mixture was poured onto crushed ice then acidified with concentrated hydrochloric acid (to pH 6). The formed solid product was collected by filtration. Crystallized from ethanol to give pale yellow crystals, yield 1.13 g (68%), m.p. 140–142 °C. *Anal*. Calculated for C_6_H_6_N_4_O_2_ (166.14): C, 43.38; H, 3.64; N, 33.72. Found: C, 43.49; H, 3.48; N, 33.59. IR, υ: 3566–3325 (OH, NH_2_), 2222 (CN), 1686 (CO). ^1^H-NMR, δ: 4.56 (s, 2H, CH_2_), 4.90 (s, 2H, NH_2_), 6.86 (s, 1H, pyrazole H-4), 10.35 (s, 1H, OH). ^13^C-NMR, δ: 38.6 (CH_2_), 117.2 (CN), 104.8, 150.4, 154.8 (pyrazole C), 172.8 (CO).

*5-Amino-3-hydroxy-1H-pyrazol-1-yl)(2,4-diamino-3-cyanothiophene-5-yl)methanone* (**7a**) and *5-amino-3-hydroxy-1H-pyrazol-1-yl)(ethyl 2,4-diaminothiophene-5-yl-3-carboxylate)methanone* (**7b**). General procedure: To a solution of compound **5** (1.66 g, 0.01 mol) in 1,4-dioxane (30 mL) containing triethylamine (1.0 mL) either malononitrile (0.66 g, 0.01 mol) or ethyl cyanoacetate (1.13 g, 0.01 mol) were added, followed by elemental sulfur (0.32 g, 0.01 mol). The whole reaction mixture, in each case was heated under reflux for 1 h then left to cool then poured onto ice/water mixture containing few drops of hydrochloric acid. The formed solid product, in each case, was collected by filtration.

Compound **7a**: Crystallized from ethanol to give yellow crystals, yield 1.90 g (72%), m.p. 166–169 °C. *Anal*. Calculated for C_9_H_8_N_6_O_2_S (264.26): C, 40.90; H, 3.05; N, 31.80; S, 12.13. Found: C, 41.11; H, 3.23; N, 32.19; S, 11.99; MS *m/z* (%): 264 (M^+^, 18%). IR, υ: 3546–3331 (OH, 3NH_2_), 2220 (CN), 1689 (CO). ^1^H-NMR, δ: 4.81, 4.83, 4.90 (3s, 6H, D_2_O exchangeable, 3 NH_2_), 6.88 (s, 1H, pyrazole H-4), 10.36 (s, 1H, D_2_O exchangeable, OH). ^13^C-NMR, δ: 116.7 (CN), 104.8, 112.8, 140.6, 147.2, 148.2, 150.2, 154.4 (pyrazole, thiophene C), 174.2 (CO).

Compound **7b**: Crystallized from ethanol to give yellow crystals, yield 2.74 g (88%), m.p. 190–193 °C. *Anal*. Calculated for C_11_H_13_N_5_O_4_S (311.32): C, 42.44; H, 4.21; N, 22.50; S, 10.30. Found: C, 42.36; H, 4.43; N, 22.79; S, 10.48; MS *m/z* (%): 311 (M^+^, 14%). IR, υ: 3556–3342 (OH, 3NH_2_), 1710, 1689 (2CO). ^1^H-NMR, δ: 1.36 (t, 3H, *J* = 7.02 Hz, CH_3_), 4.23 (q, 2H, *J* = 7.02 Hz, CH_2_), 4.79, 4.84, 4.92 (3s, 6H, D_2_O exchangeable, 3NH_2_), 6.85 (s, 1H, pyrazole H-4), 10.31 (s, 1H, D_2_O exchangeable, OH). ^13^C-NMR, δ: 16.8 (CH_3_), 44.4 (CH_2_), 105.3, 112.2, 138.9, 144.0, 146.2, 151.7, 153.8 (pyrazole, thiophene C), 163.6, 174.0 (2CO).

*(5-Amino-3-hydroxy-1H-pyrazol-1-yl)(2-amino-4,5,6,7-tetrahydrobenzo-[b]thiophen-3-yl)-methanone* (**8**). To a solution of compound **5** (1.66 g, 0.01 mol) in 1,4-dioxane (30 mL) containing triethylamine (1.0 mL), cyclohexanone (0.98 g, 0.01 mol) was added, followed by elemental sulfur (0.32 g, 0.01 mol). The whole reaction mixture was heated under reflux for 2 h then left to cool then poured onto ice/water containing few drops of hydrochloric acid. The formed solid product, in each case, was collected by filtration. Crystallized from acetic acid to give yellow crystals yield 1.95 g (70%), m.p. 220–223 °C. *Anal*. Calculated for C_12_H_14_N_4_O_2_S (278.33): C, 51.78; H, 5.07; N, 20.13; S, 11.52. Found: C, 51.94; H, 5.14; N; 20.26; S, 11.83. IR, υ: 3542–3332 (OH, 2 NH_2_), 1691 (CO). ^1^H-NMR, δ: 2.23–2.27 (2m, 8H, 4 CH_2_), 4.49, 4.80 (2s, 4H, D_2_O exchangeable, 2 NH_2_), 6.89 (s, 1H, pyrazole H-4), 10.23 (s, 1H, D_2_O exchangeable, OH). ^13^C-NMR, δ: 20.0, 23.1, 14.0, 24.6 (cyclohexene C), 109.3, 113.4, 138.6, 143.8, 144.8, 150.2, 152.9 (pyrazole, thiophene C), 160.6 (CO).

*3-(5-Amino-3-hydroxy-1H-pyrazol-1-yl)-3-oxo-2-benzalydinepropanenitrile* (**10**). To a solution of compound **5** (1.66 g, 0.01 mol) in 1,4-dioxane (40 mL) containing piperidine (0.50 mL) benzaldehyde (1.06 g, 0.01 mol) was added. The reaction mixture was heated under reflux for 3 h then left to cool. The solid product, so formed, was collected by filtration. Crystalized from ethanol to give pale yellow crystals, yield 1.27 g (50%), m.p. 180–183 °C. *Anal*. Calculated for C_13_H_10_N_4_O_2_ (254.24): C, 61.41; H, 3.96; N, 22.04. Found: C, 61.62; H, 4.29; N; 21.96. IR, υ: 3540–3341 (OH, NH_2_), 2223 (CN), 1689 (CO), 1655 (C=N). ^1^H-NMR, δ: 4.45 (s, 2H, D_2_O exchangeable, NH_2_), 6.22 (s, 1H, CH=C), 6.86 (s, 1H, pyrazole H-4), 7.28–7.33 (m, 5H, C_6_H_5_), 10.28 (s, 1H, D_2_O exchangeable, OH). ^13^C-NMR, δ: 116.3 (CN), 118.0, 120.6 (CH=C), 112.0, 124.7, 126.3, 136.2 (C_6_H_5_ C), 104.5 150.2, 152.9 (pyrazole C), 182.3 (CO).

*2-Amino-6-(5-amino-3-hydroxy-1H-pyrazol-1-yl)-4-phenyl-4H-pyran-3,5-dicarbonitrile* (**11a**) and *ethyl 2-amino-6-(5-amino-3-hydroxy-1H-pyrazol-1-yl)-5-cyano-4-phenyl-4H-pyran-3-carboxylate* (**11b**). General procedure: To a solution of compound **10** (2.54 g, 0.01 mol) in 1,4-dioxane (30 mL) containing triethylamine (0.50 mL), either malononitrile (0.66 g, 0.01 mol) or ethyl cyanoacetate (1.13 g, 0.01 mol) was added. The resulting reaction mixture in each case was heated under reflux for 6 h then the excess solvent was evaporated under reduced pressure.

Compound **11a**: Crystallized from ethanol to give yellow crystals, yield 1.92 g (60%), m.p. 193–195 °C. *Anal*. Calculated for C_16_H_12_N_6_O_2_ (320.31): C, 60.00; H, 3.78; N, 26.24. Found: C, 59.87; H, 3.53; N, 26.19; MS *m/z* (%): 320 (M^+^, 40%). IR, υ: 3555–3345 (OH, 2NH_2_), 2222, 2219 (2CN), 1648 (C=N). ^1^H-NMR, δ: 4.77, 5.21 (2s, 4H, D_2_O exchangeable, 2NH_2_), 6.40 (s, 1H, pyran H-4), 6.83 (s, 1H, pyrazole H-4), 10.32 (s, 1H, D_2_O exchangeable, OH). ^13^C-NMR, δ: 28.1 (pyran C-4), 116.8, 118.0 (2 CN), 118.4, 120.8, 126.5, 129.3, 138.3 (C_6_H_5_, pyran C), 105.2, 151.4, 154.8 (pyrazole C).

Compound **11b**: Crystallized from ethanol to give pale yellow crystals, yield 2.46 g (67%), m.p. 162–164 °C. *Anal*. Calculated for C_18_H_17_N_5_O_4_ (367.36): C, 58.85; H, 4.66; N, 19.06. Found: C, 58.66; H, 4.53; N; 19.29; MS *m/z* (%): 367 (M^+^, 18%). IR, υ: 3542–3368 (OH, NH_2_), 2222 (CN), 1687 (CO). ^1^H-NMR, δ: 1.33 (t, 3H, *J* = 6.78 Hz, CH_3_), 4.24 (q, 2H, *J* = 6.78 Hz, CH_2_), 4.65, 4.83 (2s, 4H, D_2_O exchangeable, 2NH_2_), 6.80, 6.93 (2s, 2H, pyrazole H-4, pyran H-4), 7.31–7.39 (m, 5H, C_6_H_5_), 10.36 (s, 1H, D_2_O exchangeable, OH).^ 13^C-NMR, δ: 16.8 (CH_3_), 28.9 (pyran C-4), 42.4 (CH_2_), 117.3 (CN), 118.8, 121.3, 126.2, 128.7, 138.93 (C_6_H_5_, pyran C), 105.4, 151.6, 154.6 (pyrazole C), 163.4 (CO).

*5,8-Diamino-7-cyano-2-hydroxy-6-phenyl-6,7-dihydropyrazolo[1,5-a]pyrano[2,3-d]pyrimidine* (**12a**) and *ethyl 5,8-diamino-2-hydroxy-6-phenyl-6,7-dihydropyrazolo[1,5-a]pyrano[2,3-d]pyrimidin-7-carboxylate* (**12b**). General procedure: To a solution of sodium ethoxide [prepared by dissolving sodium metal (0.23 g, 0.01 mol) in absolute ethanol (30 mL)] either compound **11a** (3.20 g, 0.01 mol) or compound **11b** (3.67 g, 0.01 mol) was added. The resulting mixture in each case was refluxed in a boiling water bath for 11 h then left to cool then poured onto ice/water mixture containing few drops of hydrochloric acid. The formed solid product in each case was collected by filtration

Compound **12a**: Crystallized from 1,4-dioxane to give yellow crystals, yield 2.34 g (73%), m.p. 256–259 °C. *Anal*. Calculated for C_16_H_12_N_6_O_2_ (320.31): C, 60.00; H, 3.78; N, 26.24. Found: C, 59.93; H, 3.94; N, 26.36; MS *m/z* (%): 320 (M^+^, 66%). IR, υ: 3528–3335 (OH, 2NH_2_), 2220 (CN), 1638 (C=C). ^1^H-NMR, δ: 4.78, 5.28 (2s, 4H, D_2_O exchangeable, 2NH_2_), 6.85, 6.92 (2s, 2H, pyrazole H-4, pyran H-4), 7.30–7.41 (m, 5H, C_6_H_5_), 10.35 (s, 1H, D_2_O exchangeable, OH). ^13^C-NMR, δ: 28.9 (pyran C-4), 116.9 (CN), 118.8, 122.6, 127.2, 129.3, 138.3, 149.2 (C_6_H_5_, pyran, pyrimidine C), 105.5, 152.0, 154.9 (pyrazole C).

Compound **12b**: Crystallized from 1,4-dioxane to give white crystals, yield 2.94 g (80%), m.p. 188–192 °C. *Anal*. Calculated for C_18_H_17_N_5_O_4_ (367.36): C, 58.85; H, 4.66; N, 19.06. Found: C, 58.93; H, 4.71; N, 19.32; MS *m/z* (%): 367 (M^+^, 65%). IR, υ: 3540–3351 (OH, 2NH_2_), 1689 (CO), 1640 (C=C). ^1^H-NMR, δ: 1.32 (t, 3H, *J* = 7.01 Hz, CH_3_), 4.24 (q, 2H, *J* = 7.01 Hz, CH_2_), 4.61, 4.88 (2s, 4H, D_2_O exchangeable, 2NH_2_), 6.52 (s, 1H, pyran H-4), 6.83 (s, 1H, pyrazole H-4), 7.28–7.37 (m, 5H, C_6_H_5_), 10.35 (s, 1H, D_2_O exchangeable, OH). ^13^C-NMR, δ: 16.9 (CH_3_), 28.9 (pyran C-4), 42.9 (CH_2_), 117.6 (CN), 118.8, 123.7, 126.8, 129.3, 138.9, 148.9 (C_6_H_5_, pyran, pyrimidine C), 105.5, 152.3, 155.1 (pyrazole C), 164.3 (CO).

*3-(5-Amino-3-hydroxy-1H-pyrazol-1-yl)-2-cyano-3-pheny-2-buten-1-one* (**14**). A mixture of compound **5** (1.66 g, 0.01 mol), acetophenone **13** (1.20 g, 0.01 mol) and ammonium acetate (1.0 g) was heated in an oil bath at 120 °C for 15 min then left to cool to room temperature. The resulting residue was then heated in ethanol (20 mL) and the formed solid product was collected by filtration. Crystallized from 1,4-dioxane to give orange crystals, yield 1.61 g (60%), m.p. 177–180 °C. *Anal*. Calculated for C_14_H_12_N_4_O_2_ (268.27): C, 62.68; H, 4.51; N, 20.88. Found: C, 62.83; H, 4.62; N, 20.76; MS *m/z* (%): 268 (M^+^, 40%). IR, υ: 3522–3341 (OH, NH_2_), 2227 (CN), 1691 (CO), 1641 (C=C). ^1^H-NMR, δ: 2.69 (s, 3H, CH_3_), 4.88 (s, 2H, D_2_O exchangeable, NH_2_), 6.88 (s, 1H, pyrazole H-4), 7.27–7.38 (m, 5H, C_6_H_5_), 10.40 (s, 1H, D_2_O exchangeable, OH). ^13^C-NMR, δ: 18.8 (CH_3_), 116.8 (CN), 118.0, 119.6 (C=C), 119.2, 120.5, 122.4, 132.6 (C_6_H_5_), 105.3, 152.6, 155.9 (pyrazole C), 163.8 (CO).

*1-(5-Amino-3-hydroxy-1H-pyrazol-1-yl)-2-thio(2-cyano-3-phenylamino-acrylonitril-3-yl)-ethanone* (**17a**), *1-(5-amino-3-hydroxy-1H-pyrazol-1-yl)-2-thio(ethyl 2-cyano-3-phenylaminoacrylat-3-yl)ethanone* (**17b**), *1-(5-amino-3-hydroxy-1H-pyrazol-1-yl)-2-thio(2-acetyl-4-phenylamino-but-3-ene-2-one-4-yl)ethanone* (**17c**) and *1-(5-amino-3-hydroxy-1H-pyrazol-1-yl)-2-thio(ethyl 2-acetyl-3-phenylaminopropenoat-3-yl)ethanone* (**17d**). General procedure: To a solution of either **6a** (0.66 g, 0.01 mol) or **6b** (1.13 g, 0.01 mol), **6c** (1.0 g, 0.01 mol) or **6d** (1.30 g, 0.01 mol) in dimethylformamide (30 mL) containing potassium hydroxide (0.56 g, 0.01 mol), phenylisothiocyanate (1.30 g, 0.01 mol) was added. The whole reaction mixture was stirred at room temperature overnight. On the next day compound **4** (1.75 g, 0.01 mol) was added with continuous stirring overnight at room temperature then poured onto ice/water containing few drops of hydrochloric acid (to pH 6). The solid product, formed in each case, was collected by filtration.

Compound **17a**: Crystallized from ethanol to give yellow crystals, yield 2.31 g (68%), m.p. 220–224 °C. *Anal*. Calculated for C_15_H_12_N_6_O_2_S (340.36): C, 52.93; H, 3.55; N, 24.69; S, 9.42. Found: C, 52.78; H, 3.63; N, 24.49; S, 9.53; MS *m/z* (%): 340 (M^+^, 15%). IR, υ: 3539–3329 (OH, NH, NH_2_), 2227, 2218 (2CN); 1688 (CO), 1634 (C=C). ^1^H-NMR, δ: 4.30 (s, 2H, CH_2_), 4.83 (s, 2H, D_2_O exchangeable, NH_2_), 6.77 (s, 1H, pyrazole H-4), 7.30–7.40 (m, 5H, C_6_H_5_), 8.30 (s, 1H, D_2_O exchangeable, NH), 10.40 (s, 1H, D_2_O exchangeable, OH).^ 13^C-NMR, δ: 38.3 (CH_2_), 116.4, 117.8 (2 CN), 120.2, 121.8, 122.8, 133.8 (C_6_H_5_), 105.2, 153.0, 155. 9 (pyrazole C), 164.2 (CO). 

Compound **17b**: Crystallized from ethanol to give yellow crystals, yield 2.86 g (74%), m.p. 195–198 °C. *Anal*. Calculated for C_17_H_17_N_5_O_4_S (387.41): C, 52.70; H, 4.42; N, 18.08; S, 8.28. Found: C, 52.68; H, 4.53; N, 18.24; S, 8.46; MS *m/z* (%): 387 (M^+^, 22%). IR, υ: 3567–3302 (OH, NH, NH_2_), 2223 (CN); 1692, 1689 (2CO), 1638 (C=C). ^1^H-NMR, δ: 1.14 (t, 3H, *J* = 6.08 Hz, CH_3_), 4.21 (q, 2H, *J* = 6.08 Hz, CH_2_), 4.32 (s, 2H, CH_2_), 4.79 (s, 2H, D_2_O exchangeable, NH_2_), 6.72 (s, 1H, pyrazole H-4), 7.28–7.38 (m, 5H, C_6_H_5_), 10.37 (s, 1H, D_2_O exchangeable, OH). ^13^C-NMR, δ: 16.4 (ester CH_3_), 38.1 (CH_2_), 41.8 (ester CH_2_), 116.8 (CN), 119.8, 122.0, 122.8, 134.0 (C_6_H_5_), 105.4, 154.2, 155.5 (pyrazole C), 160.2, 164.5 (2 CO).

Compound **17c**: Crystallized from 1,4-dioxane to give orange crystals, yield 2.99 g (80%), m.p. 145–147 °C. *Anal*. Calculated for C_17_H_18_N_4_O_4_S (374.41): C, 54.53; H, 4.85; N, 14.96; S, 8.56. Found: C, 54.64; H, 4.73; N, 14.82; S, 8.66; MS *m/z* (%): 374 (M^+^, 38%). IR, υ: 3559–3341 (OH, NH, NH_2_), 1690, 1689–1684 (3 CO), 1636 (C=C). ^1^H-NMR, δ: 2.65, 2.84 (2s, 6H, 2CH_3_), 4.40 (s, 2H, CH_2_), 4.73 (s, 2H, D_2_O exchangeable, NH_2_), 6.69 (s, 1H, pyrazole H-4), 7.30–7.39 (m, 5H, C_6_H_5_), 8.29 (s, 1H, NH), 10.39 (s, 1H, D_2_O exchangeable, OH). ^13^C-NMR, δ: 24.8, 29.4 (2 CH_3_), 38.3 (CH_2_), 120.2, 123.6, 124.2, 134.2 (C_6_H_5_), 105.2, 153.8, 155.2 (pyrazole C), 161.8, 164.8, 166.2 (3 CO).

Compound **17d**: Crystallized from ethanol to give pale yellow crystals, yield 2.26 g (56%), m.p. 120–122 °C. *Anal*. Calculated for C_18_H_20_N_4_O_5_S (404.44): C, 53.45; H, 4.98; N, 13.85; S, 7.93. Found: C, 53.55; H, 4.72; N, 13.64; S, 7.66; MS *m/z* (%): 404 (M^+^, 14%). IR, υ: 3559–3328 (OH, NH, NH_2_), 1690, 1689–1684 (3 CO), 1636 (C=C). ^1^H-NMR, δ: 1.13 (t, 3H, *J* = 7.01 Hz, CH_3_), 2.83 (s, 3H, CH_3_), 4.23 (q, 2H, *J* = 7.01 Hz, CH_2_), 4.38 (s, 2H, CH_2_), 4.81 (s, 2H, D_2_O exchangeable, NH_2_), 6.69 (s, 1H, pyrazole H-4), 7.26–7.39 (m, 5H, C_6_H_5_), 8.31 (s, 1H, D_2_O exchangeable, NH), 10.37 (s, 1H, D_2_O exchangeable, OH).^ 13^C-NMR, δ: 17.2 (ester CH_3_), 20.8 (CH_3_), 38.41 (CH_2_), 42.3 (ester CH_2_), 120.81, 122.2, 124.2, 132.8 (C_6_H_5_), 105.2, 154.42, 154.9 (pyrazole C), 160.6, 164.8, 168.3 (3 CO). 

*2-(4-(5-Amino-3-hydroxy-1H-pyrazol-1-yl)-3-phenylthiazol-2(3H)-ylidene)-malononitrile* (**18a**), *ethyl 2-(4-(5-aino-3-hydroxy-1H-pyrazol-1-yl)-3-phenylthiazol-2(3H)-ylidene)-2-cyanoacetate* (**18b**), *2-(4-(5-amino-3-hydroxy-1H-pyrazol-1-yl)-3-phenylthiazol-2(3H)-ylidene)pentane-2,4-dione* (**18c**) and *ethyl 2-(4-(5-amino-3-hydroxy-1H-pyrazol-1-yl)-3-phenylthiazol-2(3H)-ylidene)-3-oxo-butanoate* (**18d**). General procedure: To a suspension of either compound **17a **(3.40 g, 0.01 mol), **17b** (3.87 g, 0.01 mol), **17c** (3.74 g, 0.01 mol) or **17d** (4.04 g, 0.01 mol) in sodium ethoxide solution [prepared by dissolving metallic solvent (0.64 g, 0.01 mol) in absolute ethanol (30 mL)] was boiled in a boiling water bath for 8 h. The reaction mixture was left to cool then poured onto ice/water containing few drops of hydrochloric acid. The formed solid product was collected by filtration.

Compound **18a**: Crystallized from ethanol to give yellow crystals, yield 2.48 g (77%), m.p. 260–263 °C. *Anal*. Calculated for C_15_H_10_N_6_OS (322.34): C, 55.89; H, 3.13; N, 26.07; S, 9.95. Found: C, 55.79; H, 3.43; N, 26.22; S, 9.76; MS *m/z* (%): 322 (M^+^, 80%). IR, υ: 3549–3338 (OH, NH_2_), 2225, 2215 (2CN); 1637 (C=C). ^1^H-NMR, δ: 4.88 (s, 2H, D_2_O exchangeable, NH_2_), 6.79, 6.93 (2s, 2H, pyrazole H-4, thiazole H-5), 7.32–7.43 (m, 5H, C_6_H_5_), 10.40 (s, 1H, D_2_O exchangeable, OH). ^13^C-NMR, δ: 116.8, 118.3 (2 CN), 119.4, 122.0, 122.6, 134.0, 144.2, 152.9, 153.1 (C_6_H_5_, thiazole C), 132.1, 138.9 (C=C), 105.2, 153.0, 155. 9 (pyrazole C).

Compound **18b**: Crystallized from ethanol to give buff crystals, yield 2.82 g (80%), m.p. 188–191 °C. *Anal*. Calculated for C_17_H_15_N_5_O_3_S (369.40): C, 55.27; H, 4.09; N, 18.96; S, 8.68. Found: C, 55.59; H, 4.37; N, 18.82; S, 8.56; MS *m/z* (%): 369 (M^+^, 77%). IR, υ: 3576–3326 (OH, NH_2_), 2220 (CN), 1686 (CO), 1638 (C=C). ^1^H-NMR, δ: 1.13 (t, 3H, *J* = 7.11 Hz, CH_3_), 4.24 (q, 2H, *J* = 7.11 Hz, CH_2_), 4.83 (s, 2H, D_2_O exchangeable, NH_2_), 6.70, 6.83 (2s, 2H, pyrazole H-4, thiazole H-5), 7.32–7.39 (m, 5H, C_6_H_5_), 10.35 (s, 1H, D_2_O exchangeable, OH). ^13^C-NMR, δ: 16.8 (ester CH_3_), 40.8 (ester CH_2_), 116.6 (CN), 120.6, 122.3, 124.8, 134.0, 143.8, 152.4, 153.8 (C_6_H_5_, thiazole C), 132.1, 138.0 (C=C), 105.6, 154.2, 155.3 (pyrazole C), 164.8 (CO).

Compound **18c**: Crystallized from 1,4-dioxane to give pale yellow crystals, yield 2.49 g (70%), m.p. 199–202 °C. *Anal*. Calculated for C_17_H_16_N_4_O_3_S (356.40): C, 57.29; H, 4.52; N, 15.72; S, 9.00. Found: C, 57.49; H, 4.58; N, 16.02; S, 9.04; MS *m/z* (%): 356 (M^+^, 22%). IR, υ: 3545–3322 (OH, NH_2_), 1692, 1689 (2 CO), 1634 (C=C). ^1^H-NMR, δ: 2.59, 2.82 (2s, 6H, 2CH_3_), 4.43 (s, 2H, D_2_O exchangeable, NH_2_), 6.63, 6.93 (2s, 2H, pyrazole H-4, thiazole H-5), 7.28–7.35 (m, 5H, C_6_H_5_), 10.31 (s, 1H, D_2_O exchangeable, OH). ^13^C-NMR, δ: 24.8, 28.6 (2 CH_3_), 120.8, 123.0, 126.9, 134.2, 143.8, 153.0, 154.4 (C_6_H_5_, thiazole C), 132.8, 138.9 (C=C), 105.3, 154.0, 154.8 (pyrazole C), 162.7, 164.6 (2 CO).

Compound **18d**: Crystallized from ethanol to give yellow crystals, yield 2.66 g (69%), m.p. 221–224 °C. *Anal*. Calculated for C_18_H_18_N_4_O_4_S (386.42): C, 55.95; H, 4.70; N, 14.50; S, 8.30. Found: C, 56.27; H, 4.67; N, 14.39; S, 8.52; MS *m/z* (%): 386 (M^+^, 40%). IR, υ: 3541–3339 (OH, NH_2_), 1688, 1680 (2 CO), 1631 (C=C). ^1^H-NMR, δ: 1.14 (t, 3H, *J* = 6.55 Hz, CH_3_), 2.82 (s, 3H, CH_3_), 4.21 (q, 2H, *J* = 6.55 Hz, CH_2_), 4.59 (s, 2H, D_2_O exchangeable, NH_2_), 6.55, 6.80 (2s, 2H, pyrazole H-4, thiazole H-5), 7.28–7.35 (m, 5H, C_6_H_5_), 10.24 (s, 1H, D_2_O exchangeable, OH).^ 13^C-NMR, δ: 16.8 (ester CH_3_), 24.5 (CH_3_), 42.0 (ester CH_2_), 121.3, 123.1, 126.6, 134.0, 144.0, 153.0, 153.8 (C_6_H_5_, thiazole C), 133.0, 138.9 (C=C), 105.1, 154.2, 154.4 (pyrazole C), 163.2, 164.8 (2 CO).

## 4. Conclusions

The aim of this work was to synthesize a series of new pyrazole derivatives. The key intermediate for most of these molecules was *N*'-(2-chloroacetyl)-2-cyanoacetohydrazide (**3**), which underwent ready cyclization to give 1-(5-amino-3-hydroxy-1*H*-pyrazol-1-yl)-2-chloroethanone (**4**). The anti-tumor evaluations of the newly synthesized pyrazole derivatives showed that among the tested compounds 5,8-diamino-7-cyano-2-hydroxy-6-phenyl-6,7-dihydropyrazolo[1,5-a]pyrano[2,3-d]-pyrimidine (**12a**), 2-(4-(5-amino-3-hydroxy-1H-pyraol-1-yl)-3-phenylthiazol-2(3H)-ylidene)-malononitrile (**18a**) and 2-(4-(5-amino-3-hydroxy-1H-pyrazol-1-yl)-3-phenylthiazol-2(*3H*)-ylidene)-pentane-2,4-dione (**18c**) showed the best results, exhibiting the highest inhibitory effects towards the three tumor cell lines, which were higher than that of the reference doxorubicin. Such high cytotoxicity of **12a**, **18a** and **18c** is attributed to the presence of strong electron withdrawing groups together with their solubility in polar solvents.
